# Controlling the degradation kinetics of porous iron by poly(lactic-*co*-glycolic acid) infiltration for use as temporary medical implants

**DOI:** 10.1038/srep11194

**Published:** 2015-06-09

**Authors:** Abdul Hakim Md Yusop, Nurizzati Mohd Daud, Hadi Nur, Mohammed Rafiq Abdul Kadir, Hendra Hermawan

**Affiliations:** 1 Medical Devices Technology Group (MediTeg), Faculty of Biosciences and Medical Engineering, Universiti Teknologi Malaysia, Johor Bahru, 81310, Malaysia; 2 Center for Sustainable Nanomaterials, Ibnu Sina Institute for Scientific and Industrial Research, Universiti Teknologi Malaysia, Johor Bahru, 81310, Malaysia; 3 Dept. of Mining, Metallurgical and Materials Engineering & CHU de Québec Research Center, Laval University, Quebec City, G1V 0A6, Canada

## Abstract

Iron and its alloy have been proposed as biodegradable metals for temporary medical implants. However, the formation of iron oxide and iron phosphate on their surface slows down their degradation kinetics in both *in vitro* and *in vivo* scenarios. This work presents new approach to tailor degradation behavior of iron by incorporating biodegradable polymers into the metal. Porous pure iron (PPI) was vacuum infiltrated by poly(lactic-*co*-glycolic acid) (PLGA) to form fully dense PLGA-infiltrated porous iron (PIPI) and dip coated into the PLGA to form partially dense PLGA-coated porous iron (PCPI). Results showed that compressive strength and toughness of the PIPI and PCPI were higher compared to PPI. A strong interfacial interaction was developed between the PLGA layer and the iron surface. Degradation rate of PIPI and PCPI was higher than that of PPI due to the effect of PLGA hydrolysis. The fast degradation of PIPI did not affect the viability of human fibroblast cells. Finally, this work discusses a degradation mechanism for PIPI and the effect of PLGA incorporation in accelerating the degradation of iron.

Biodegradable metals have been studied as new potential materials for load-bearing temporary medical implants where they support the healing process of diseased tissue and progressively degrade thereafter^1^. Gradual transfer of loads to the healing tissue and non-necessity of a second removal surgery make biodegradable metals favorable alternatives to the existing metal implants used for temporary interventions^2^,^3^. Biodegradable metals have been proposed for various temporary implants including endovascular stents and bone pins and screws, where bulk metal form is required^4^.

Recently, porous biodegradable metals have also been viewed as potential materials for hard tissue scaffolds^5^,^6^. Porous structure is needed to accommodate cell proliferation, tissue formation and for diffusion of nutrients to and metabolites out from the cell/scaffold construct^7^,^8^,^9^. Porous biodegradable metal scaffolds have been made mostly from magnesium and its alloys^10^,^11^,^12^,^13^,^14^. Compared to its solid structure, degradation of porous magnesium resulted in a lower pH change, slower H_2_ evolution and slower decrement of compressive yield strength in immersion tests using simulated body fluid (SBF)^15^. Its porous structure was proven to play important role in cell growth and proliferation due to higher surface contact area and interconnectivity within the pores which lead to the greater cell spreading^16^,^17^. More recently, porous iron was also introduced as material for hard tissue scaffolds^18^,^19^,^20^. *In vitro* cytotoxicity assessment on porous Fe-Mg, pure iron and Fe-CNTs manufactured by replication method showed a small proliferation effect towards osteoblast^19^. Degradation products of iron and its alloys were found non-toxic and cytocompatible to various cell types including endothelial^21^,^22^, fibroblast^6^,^23^, smooth muscle^22^,^24^, umbilical vein endothelial cells^25^ and mesenchymal^6^ and also haemocompatible with human blood^22^,^24^,^26^,^27^.

However, degradation rate of porous iron is still considered too slow and unmatched with the tissue healing period^23^,^28^,^29^. The formation of iron oxides have been identified as the main constraint for a faster degradation but it is inevitable as the availability oxygen is necessary to commence iron degradation^23^,^30^. The dense degradation products such as iron-hydroxides, iron-carbonates and iron-phosphate layers greatly hinder oxygen transport to the fresh iron surface^27^,^30^,^31^. Attempts to accelerate the degradation kinetics of iron have been explored through alloying, thermomechanical treatment and by making composite of iron with bioceramics^32^,^33^. Electroformed iron with finer grain sizes and preferential textures was found to have a slight increase of corrosion rate^34^. Iron was coated with micro-patterned Au disc arrays and produced a more uniform corrosion with an almost four times higher degradation rate than the uncoated ones^35^. The composite of iron with Fe_2_O_3_ created more phase/grain boundaries which acted as active sites for accelerating degradation^22^.

Above all, the reason for slow degradation kinetics of iron is the formation of passive iron oxide and phosphate layers. Acidic condition has been known to greatly escalate the dissolution and solubility of iron degradation products^36^,^37^. Therefore, having local acidity on the iron surface, such as from hydrolysis of polymers, could be an alternative way to dissolve the degradation layer. Coating of biodegradable polymers, such as poly(lactic acid), poly(caprolactone) and poly(lactic-*co*-glycolic acid) on bulk magnesium and its alloys, in an attempt to slow down their degradation, has generally turn to the opposite results^38^,^39^,^40^,^41^,^42^. Among synthetic biodegradable polymers, poly(lactic-*co*-glycolic acid) or PLGA degrades quite rapidly. It takes 1-2 months for a complete degradation depending on the ratio of lactic to glycolic acids^43^. PLGA has also shown its cytocompatibility as it supports osteoblast attachment, growth, and function both *in vitro* and *in vivo*^44^,^45^,^46^,^47^. This work presents a new approach to accelerate the degradation rate of iron by infiltrating and coating PLGA into the three dimensional porous structure of iron. It is expected that the hydrolysis of PLGA will dissolve the adhered iron oxide or phosphate layers and thus promotes overall iron degradation. A solid understanding of the degradation mechanism of this iron-PLGA composite structure will allow a better formulation to control porous iron degradation.

## Results and Discussions

### Microstructure and interfacial interaction

**Figure 1:**
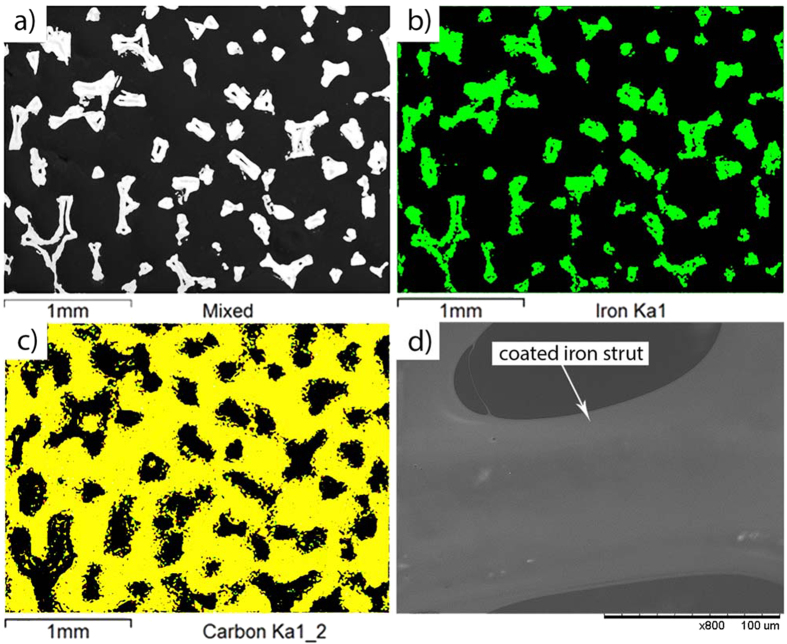
Microstructure of PCPI and PIPI samples: (**a**) SEM image of iron struts (white) and PLGA (black), (**b**,**c**) EDS maps of iron (green) and carbon (yellow) in a PIPI sample; and (**d**) SEM image of an interface between PLGA and iron strut in a PCPI sample.

Figure 1a presents backscattered electron mode SEM image showing the evidence of uniform infiltration of PLGA (dark area) into the porous iron structure (white area) in PIPI sample. This infiltration is further confirmed by the EDS elemental map in Fig. 1b,c, where carbon in PLGA (yellow colored area) occupied porous region surrounding the iron struts (green colored area). Figure 1d shows a secondary electron mode SEM image of PCPI where the PLGA appeared to uniformly covers the iron struts as indicated by the uniform greyish layer. The absence of void in the PIPI samples indicates an optimum vacuum infiltration parameter was reached.

**Figure 2:**
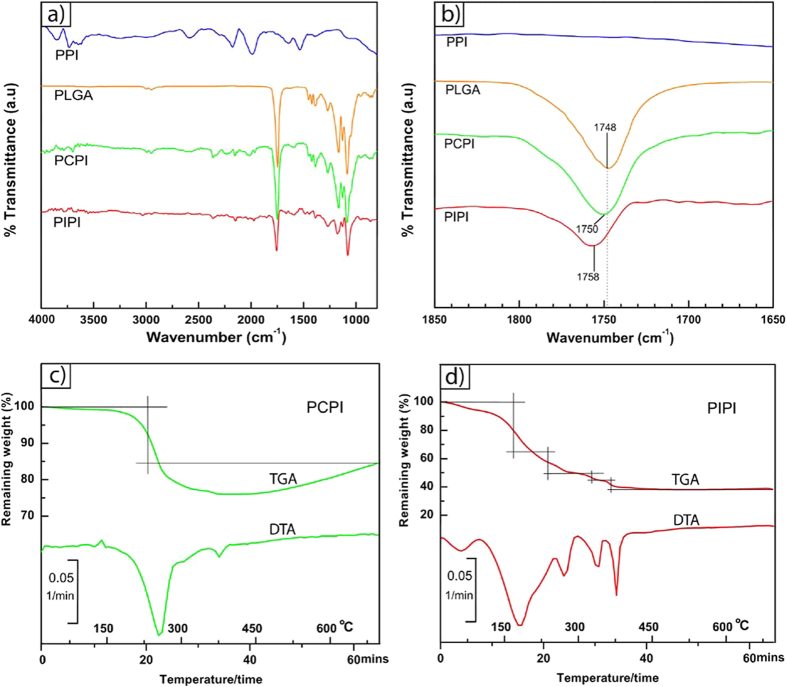
IR spectra and TGA/DTA curves of PPI, PLGA, PCPI and PIPI samples: (**a**) IR spectra of all samples, (**b**) IR spectra in the region of ≈1750 cm^−1^ showing the shifting in the C=O carbonyl group peaks, (**c**,**d**) TGA and DTA curves of PCPI and PIPI samples, respectively.

Figure 2 shows evidence of strong interfacial interaction between PLGA iron struts in the samples. The IR spectra indicates an intense peak at 1748 cm^−1^ of PLGA spectrum assigned to carbonyl (C=O) stretching, one of the characteristic peaks for PLGA (Fig. 2a). A shifting of the carbonyl group peak is observed from 1748 cm^−1^ to 1750 cm^−1^ in PCPI and to 1758 cm^−1^ in PIPI (Fig. 2b). This shifting indicates that the interfacial interaction between iron atoms and carbonyl molecules tends to disrupt the self-association between carbonyl molecules in PLGA^48^,^49^,^50^,^51^. Besides, iron is heavier in atomic weight compared to those of C and O atoms, and the iron-carbonyl molecules interactions make the C=O bond more restricted and larger force is necessary to make C=O bond vibrate. This consequently leads to the increase in frequency and wavenumber of the C=O bond^52^. Further supports from TGA-DTA results shows a trend of gradual mass loss of PLGA the PIPI associated with an endothermic process compared to that in PCPI, which conversely has a steeper slope of mass loss (Fig. 2c,d). This indicates a stronger interfacial interaction between PLGA and iron in PIPI than in PCPI and this affects different degradation profile of PCPI and PIPI as will be discussed later.

**Figure 3:**
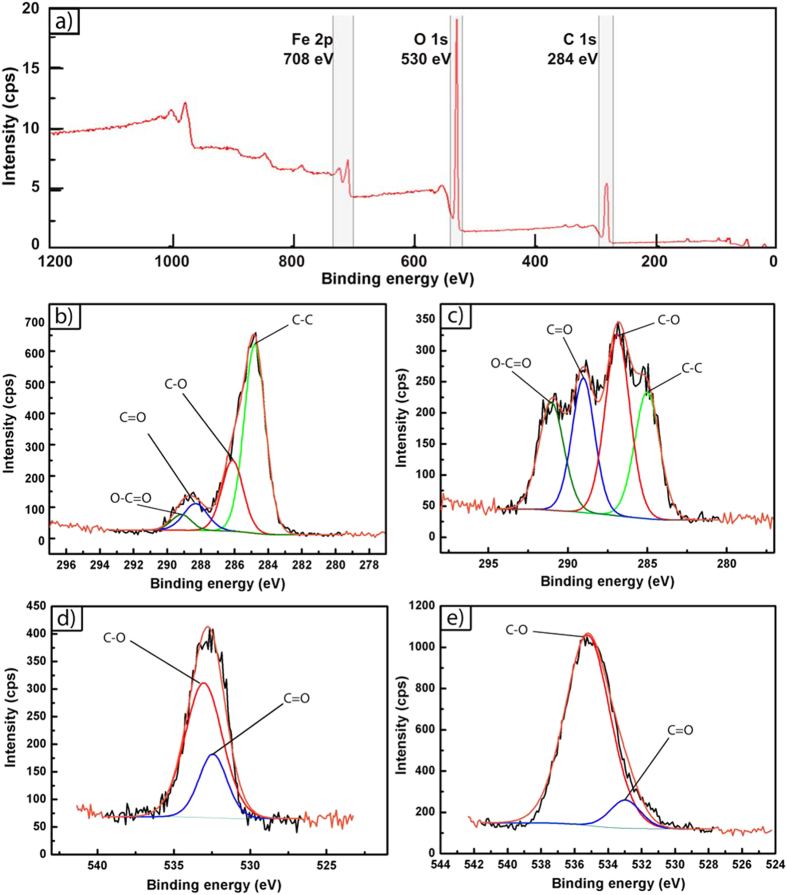
XPS spectra of PIPI sample: (**a**) the survey spectra showing the peaks of Fe, C and O, (**b**,**c**) high resolution spectra of C_1s_ before and after infiltration, respectively, (**d**,**e**) high resolution spectra of O_1s_ before and after infiltration, respectively.

Figure 3a shows XPS survey spectra of the PIPI sample showing the presence of Fe_2p_, O_1s_ and C_1s_ peaks. High resolution spectra of C_1s_ and O_1s_ for PLGA and PIPI (Fig. 3a–e) indicate a slight increase of binding energy for C=O component in PIPI with respect to pure PLGA. As detailed in Table 1, the binding energy increased from 288.31 eV to 289.03 eV in C_1s_ component. This suggests that the carbonyl groups in PIPI could be interconnected via intermolecular hydrogen bonding and once deposited on iron surface, the bonding was disrupted and became weaker and consequently gave rise on the C=O binding energy^50^. This is also consistent with the ATR-FTIR results which demonstrate an upward shifting of C=O stretching from 1748 to 1758 cm^−1^ (Fig. 2b). A similar trend was also observed in O_1s_ peak of the C=O component where the binding energy exhibit an increase from 532.44 to 532.97 eV. The higher electron withdrawing capacity of iron removed more valence electrons of carbon atom in the carbonyl group, leading to the enhancement of effective nuclear charge experienced by the core-electron and subsequently increasing the electron binding energy^52^. The C_1s_ peak intensity is expected to increase when organic molecules interact with a surface^52^,^53^. This is evidently seen for C-O, O-C=O and C=O peaks after absorption of PLGA on iron surface in PIPI. The exceptional is only for the C-C peaks postulating that the C-C did not involve in the iron-PLGA surface interaction.

### Mechanical property

**Figure 4:**
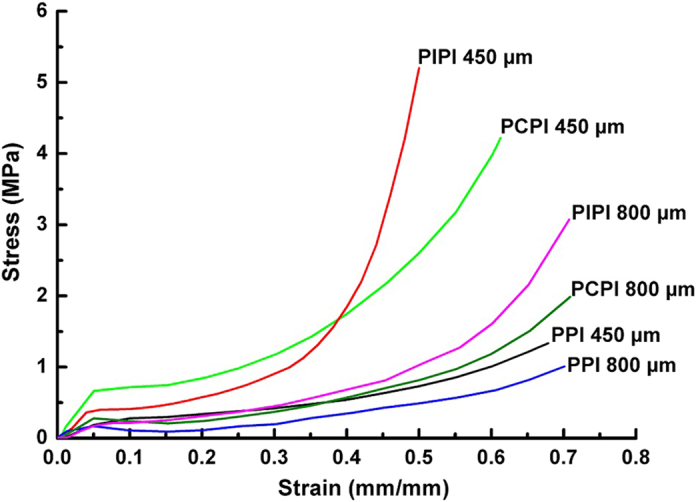
Compressive stress-strain curves of PPI, PCPI and PIPI samples.

Figure 4 shows typical compressive stress-strain curves of PPI, PCPI and PIPI samples with two different pore sizes (450 and 800 μm). It can be seen that the incorporation of PLGA into the porosity of iron generally increases the strength of both PCPI and PIPI samples. Plastic deformation occurred after the first maximum for all cases which are consistent with other studies^10^,^54^,^55^. In addition, it shows that all samples exhibit smooth curves that are typical characteristics of ductile metallic foam^56^. Elastic deformation portions of all specimens are in the range of 0.03–0.05 strain within 0.3–0.6 MPa stress values. These small ranges imply that all samples exhibited low elastic deformation at low compressive stress before undergoing large plastic deformation. Interestingly, the compressive strength of all samples is in the range of that of cancellous bone (0.2–10 MPa)^57^,^58^. This within-the-range strength values are of vital property for a synthetic material to be applied as a bone graft.

An obvious difference among the PPI, PCPI and PIPI curves was observed on the plateau region and densification stage. The extended plateau regions of PPI samples were evidently seen and ensue in relatively longer strain range (0.1–0.25 strain) compared to those of PCPI (0.1–0.15 strain) and PIPI (0.08–0.14 strain). The plateau region refers to the possible events of buckling, yield and fractures of cell walls^56^,^59^. These occurrences predominantly took place in PPI as there had been no impeding feature (infiltrated PLGA) adjacent to the walls that made it free to deform in a manner of buckling, yielding or fracture. Upon entering the densification region, buckling of the struts was relatively more difficult to take place as higher stresses were required to give a significant displacement on the iron strut and this phenomenon is prominently taken place in PCPI and PIPI due to the presence of adjacent impregnated PLGA. In the PCPI and PIPI samples with smaller pore size (450 μm), it is obvious that after a short range of plateau region, the samples tended to be densified as depicted by steeper slopes of the curves within 0.38–0.6 strain. In comparison, these increments were more prominent in PIPI than PCPI indicated by a steeper slope of the PIPI. This was an expected result since the PLGA impregnation in the PIPI made the foam more compact and become denser as a whole.

The PCPI possesses slightly higher strength compared to PIPI and PPI (Table 2). Meanwhile, samples having larger pore size (800 μm) exhibited lower strength compared to those with smaller pore size (450 μm). The effect of pore size is observed on the strength and modulus where all 450 μm samples are superior to the 800 μm samples. This is due to the fact that smaller pore size can bear greater compressive load as cell walls are thicker than that in larger pore size^55^. This could lead to a higher modulus of elasticity and peak stress (compressive strength). Meanwhile porosity also influences the mechanical properties of metal foam as the yield strength and subsequently compressive strength may increase due to lower porosity^10^,^55^. This phenomenon has been revealed in this study as all the 450 μm samples having lower porosity of 88% compared to that of 800 μm samples (92% porosity) leading to higher yield and compressive strength in all 450 μm samples. PIPI samples possess slightly higher toughness compared to that of PCPI. This is evidently seen by higher area bounded by the PIPI curves in the densification region. Larger amount of PLGA in PIPI makes it more ductile and consequently enable it to absorb more energy before it undergoes fracture. Conclusively, the incorporation of the PLGA on the iron increases its toughness as a whole.

### Degradation behavior

**Figure 5:**
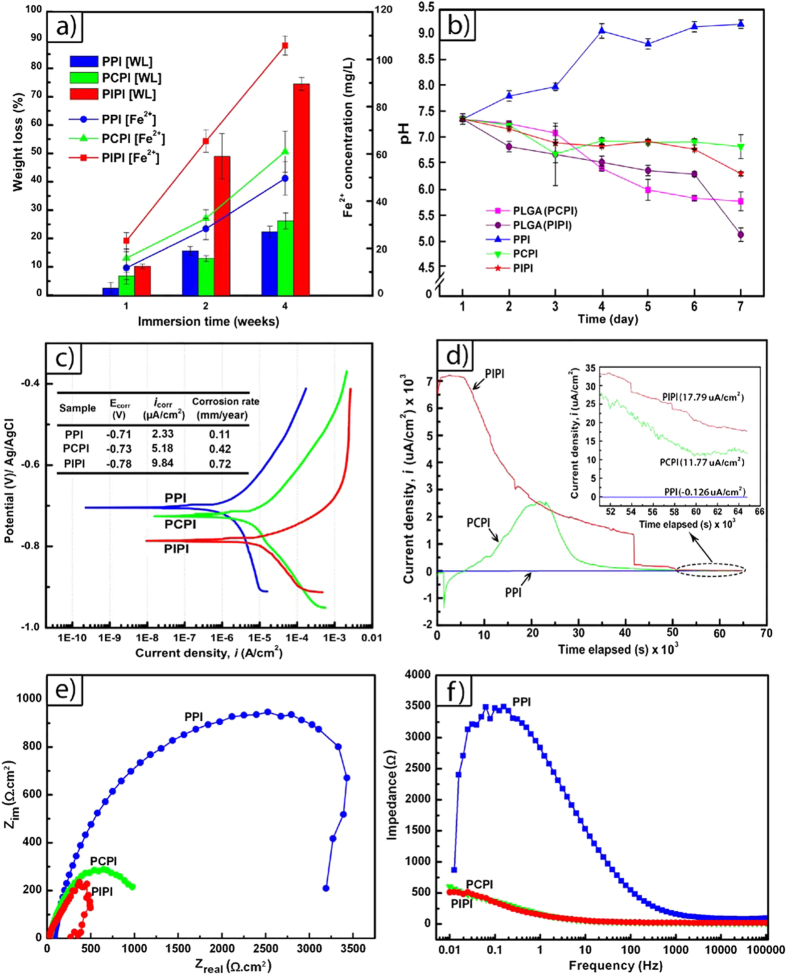
Degradation assessment results: (**a**) Weight loss (WL) and Fe^2+^ concentration of PPI, PCPI and PIPI samples after immersion in PBS for 1, 2, and 4 weeks, (**b**) pH measurement data up to 7 days immersion in PBS. (**c**) PDP **c**urves of the samples and their corresponding corrosion parameters, (**d**) PSP curves after 7 days immersion in PBS, (**e**,**f**) Nyquist and Bode plots of the samples from the EIS after 2 h exposure to PBS solution.

Figure 5 shows results of five different assessment of degradation behavior of PPI, PCPI and PIPI samples. Based on weight loss measurement of the degraded samples at week 1, 2 and 4 of immersion in PBS (Fig. 5a), a prediction for yearly degradation rate indicates that PIPI experienced the highest Fe^2+^ concentration and degradation rate (6.42 mm/year) followed by PCPI (0.76 mm/year) and PPI (0.33 mm/year). Higher weight loss in PIPI can be attributed to the rapid degradation (hydrolysis) of PLGA. In association with immersion test, pH measurement shows a decreasing value from 7.5 to 5.82 and to 5.18 for pure PLGA samples with equivalent weight to that in PCPI and in PIPI samples (Fig. 5b). The TGA-DTA results (Fig. 2c,d) shows that only ~15% carbon (represent pure PLGA) remained in the PCPI while ~55% carbon remained in the PIPI. The weight of the pure PLGA (PIPI) samples was actually higher than that of pure PLGA (PCPI). Different weight of PLGA leads to different amount of acidic species released during hydrolysis and consequently give slightly different pH profile between PLGA (PCPI) and PLGA (PIPI). Meanwhile, the degradation of pure iron increased pH from 7.4 to 9.22 after 7 days immersion as it has been known that its degradation generally produces OH^−^ ions^60^. The incorporation of PLGA in PCPI and PIPI promoted a decreasing trend of the pH value where it lowered down to 6.87 for PCPI and 6.35 for PIPI at day 7. This trend can be attributed to the pH compensation of the basic product of iron degradation by acidic hydrolysis products of PLGA.

As shown by the potentiodynamic polarization curves in Fig. 5c, corrosion current density (i_corr_) of the samples increases in order of PPI < PCPI < PIPI. The PIPI possess the highest corrosion rate (0.72 mm/year) compared to that of PCPI (0.42 mm/year) and PPI (0.11 mm/year), a similar trend of degradation rate as that for immersion test. The corrosion potential of PIPI is the lowest among others indicating higher corrosion susceptibility for the PIPI. The shifting of corrosion potentials to more negative values indicates that the polarization are mostly occurred at the cathode and hence the corrosion rates of iron in PCPI and PIPI are cathodically controlled^61^,^62^. Furthermore, the acidic species released during PLGA hydrolysis within the PIPI created local acidic environment that lead to reduction of hydrogen ion, H^+^ as cathodic reaction, together with common oxygen reduction. The exchange of i_*corr*_ in H^+^ reduction is much higher than that in oxygen reduction, and results in higher i_*corr*_ and consequently the corrosion rate^63^. The potentiostatic polarization curves in Fig. 5d demonstrate a constant voltage of −0.5 V for all samples^61^. After 7 days of immersion, PPI current density decreases to −0.126 μA/cm^2^ from 2.33 μA/cm^2^ at day 1 postulating that the passive oxide layer resided on the iron surface and hence slow down the degradation rate of iron. PCPI and PIPI had the constant current densities of 11.77 and 17.79 μA/cm^2^ respectively. These values are still much higher than those at day 1, which are 5.18 and 9.84 μA/cm^2^ for PCPI and PIPI respectively. This indicates that the degradation rate of iron in PCPI and PIPI have been accelerated in the presence of PLGA.

Further analysis on the impedance behavior at the iron/PLGA layer interface by EIS (Figs. 5e,f) shows that the obtained impedance diagrams are not perfectly semicircles attributed to a frequency dispersion^64^,^65^. By using Simplified Randle Cell method, the interface was characterized by the charge-transfer resistance (R_ct_) or so-called polarization resistance (R_p_) and by the double layer capacitance (C_dl_) whose values depend on the increase of wetted metal area. PPI sample showed a larger R_p_ (Fig. 5e) indicating a presence of an inhibition or passive layer. Iron oxides or phosphate layers were found to form on iron immersed in *in vitro* simulated body fluids and also *in vivo*^23^,^30^,^31^. The C_dl_ of the PPI is relatively low compared to those of PCPI and PIPI (Table 3) indicating a decrease of wetted area on the PPI surface due to the formation of degradation products.

The impedance tends to decrease with the incorporation of the PLGA onto the iron surface as shown by PCPI and PIPI samples. The cause of this decrement is related to the increase of disconnected area at the metal/PLGA layer interface could be due to the delamination of the PLGA layer. Consequently, the delamination would allow more penetration of the electrolyte onto the iron surface. The total impedance, |Z| measured at the lowest frequency, f = 0.01 Hz could be a general indicator of the inhibiting capacity of any layers or protective films that reside on the metal surface at a predetermined time. Referring to Fig. 5f, even being coated by the PLGA layer, the total impedances of the PCPI and PIPI are still relatively lower than that of PPI indicating a good permeation of the electrolyte through the micropores in the PLGA matrix.

### Surface property

**Figure 6:**
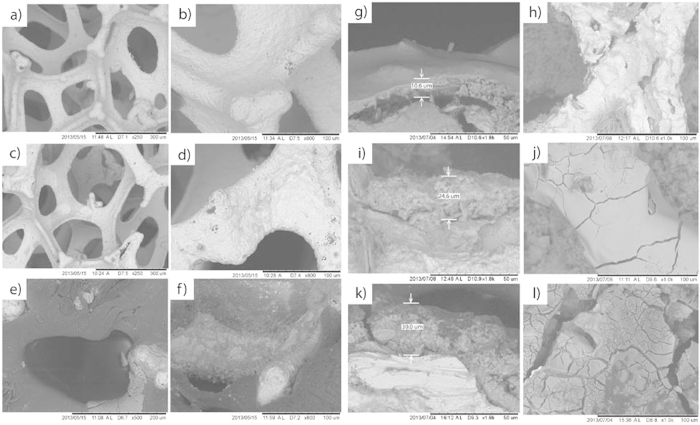
SEM micrographs of: (**a**,**b**) PPI, (**c**,**d**) PCPI and (**e**,**f**) PIPI samples after 1 week of degradation. Degradation layer and surface morphology of (**g**,**h**) PPI, (**i**,**j**) PCPI and (**k**,**l**) PIPI samples after 4 weeks of degradation.

Figure 6 shows SEM micrographs of PPI, PCPI and PIPI samples after 1 and 4 weeks of degradation. Pits are evidently observed on the struts attributed to the iron dissolution through anodic reaction as well as diffusion of oxygen molecules onto the iron surface via cathodic reaction. In PIPI sample, degradation of PLGA was seen to cover the struts and as the effect cracks were also found on the struts (Fig. 6e,f). After 4 weeks, some PPI’s struts experienced rigorous break and larger pit formation (Fig. 6g,h). Cracks were observed on the PCPI’s struts (Fig. 6i,j) as well as on the PIPI’s struts (Fig. 6k,l). For all samples, the interface between the substrate and degradation layer looks quite rough. The degradation in the PIPI is evidently much more pronounced as the degradation layer is about three times thicker than that of PPI (Fig. 6g–k).

**Figure 7:**
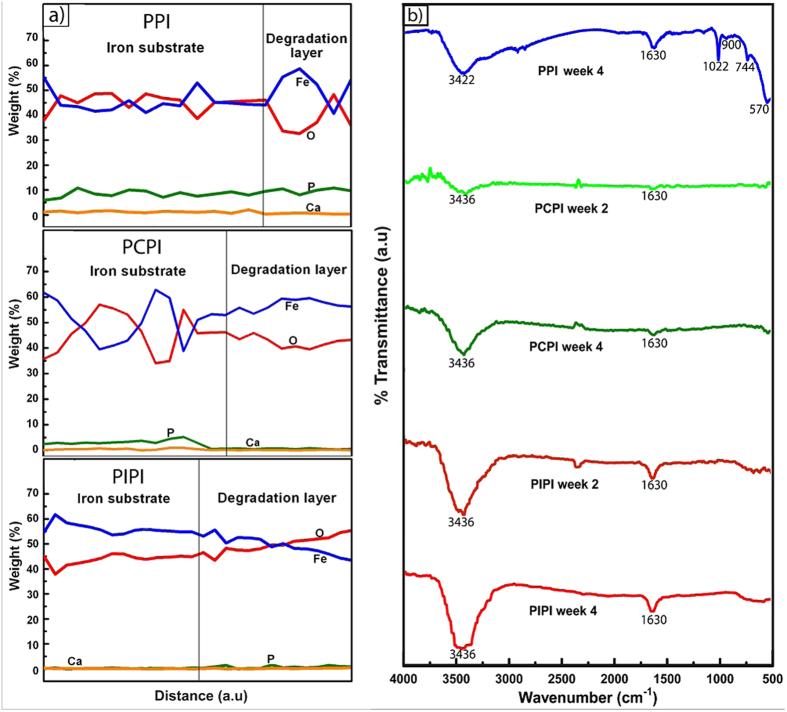
Assessment on degradation layer: (**a**) EDS elemental profiles across the iron substrate and degradation layer, (**b**) IR spectra of PPI, PCPI and PIPI degradation products after 2 and 4 weeks of degradation.

Figure 7 shows EDS elemental profile across the degradation layer and IR spectra of degradation product of PPI, PCPI and PIPI samples after 2 and 4 week. The elemental profile (Fig. 7a) shows that the degradation layer of PPI contains Fe, O, Ca and P while only minor amount of Ca and P found in PCPI and PIPI. Although the presence of the inorganic elements, especially P, were just about 8% in the degradation layer of PPI, it may form phosphate layer on the strut that could further inhibit degradation of iron by hindering oxygen diffusion. There were fewer depositions of the inorganic ions in PCPI and PIPI. The diffusion of these ions may have been inhibited by the PLGA layer and this could be attributable to the lower concentration gradient of the basic PO_4_^3−^ ions in the vicinity of the acidic PLGA layer. This hypothesis is consistent with the findings reported by previous studies^63^,^66^. The concentration of inorganic salts in the electrolyte significantly influences the solubility of oxygen which could approach zero in a highly-concentrated solution^67^. As the concentration of salts in the aqueous interlayer between substrate and PLGA has lowered due to the little diffusions of inorganic ions, there would be more oxygen diffusion taking place into the layer which could then trigger further degradation of iron.

The IR spectra (Fig. 7b) indicate the presence of five peaks on PPI (3422 cm^−1^, 1022 cm^−1^, 900 cm^−1^, 744 cm^−1^ and 570 cm^−1^), and only two peaks on PCPI and PIPI (3436 cm^−1^ and 1630 cm^−1^). A broad peak at 3422 cm^−1^ on PPI indicates the existence of *b*-FeOOH or akaganéite in the degradation products^68^,^69^. A strong peak at 1630 cm^−1^ connotes an O-H stretching due to adsorbed water in the degraded pure iron^70^,^71^,^72^. Another strong peak at 1022 cm^−1^ implies the existence of γ-FeOOH or lepidocrocite. Two medium intensity peaks at 900 cm^−1^ and 744 cm^−1^ ascertain the existence of α-FeOOH (goethite) and γ-FeOOH (lepidocrocite), respectively^73^,^74^,^75^. Apart from that, a relatively weak peak at 570 cm^−1^ reveals Fe-O stretching vibration in magnetite, Fe_3_O_4_^76^. It can be recapitulated that the absorption band at higher wave number region is due to OH stretching whereas at lower wave number, the absorption is owing to Fe-O lattice vibration. On the PCPI and PIPI spectra, a strong and broad peak at 3436 cm^−1^ indicates the O-H stretching vibration of hydroxyl end groups in the degradation products^77^ whilst a strong and sharp peak at 1630 cm^−1^ is assigned to O-H stretching due to the presence of water molecules in the degradation products (hydration). The band at 3436 cm^−1^ signifies the presence of the hydroxyl group (OH) proving the hydrolysis of PLGA in the PCPI and PIPI. It is known that the ester group in the PLGA hydrolyzed into OH and carboxyl (RCOOH) end group through the hydrolysis which leads to an overall increase of OH groups in the degrading PLGA^77^,^78^. Intensity of the 3436 cm^−1^ peaks at week 4 is relatively higher for both of PCPI and PIPI, compared to those at week 2. This obviously indicates that more hydrolysis had taken place in samples at week 4 as OH groups increased with the progression of immersion time. In addition, it can be postulated here that the degradations of iron in the PCPI and PIPI are greatly proportional to the rate of the PLGA hydrolysis.

**Figure 8:**
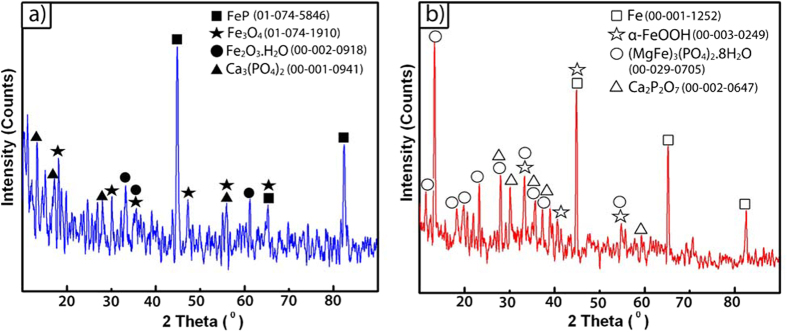
XRD patterns of the degraded: (**a**) PPI **a**nd (**b**) PIPI samples. Note: PDF numbers are given in the bracket.

The XRD patterns on the surface of PPI and PIPI samples after 4 weeks of degradation (Fig. 8) identify the presence of FeP, Fe_3_O_4_, Fe_2_O_3_.H_2_O, Ca_3_(PO_4_)_2_, Ca_2_P_2_O_7_, α-FeOOH and (MgFe)_3_(PO_4_)_2_.8H_2_O. The low solubility Fe_3_O_4_, which was formed at the early corrosion process due to insufficient oxygen supply to the deposited Fe(OH)_2_, resides at the innermost degradation layer in contact with the iron substrate and impedes further degradation of iron^60^. Similarly, the FeP which has low solubility in water also impedes the degradation by hindering oxygen transport onto iron surface^60^. The Ca_3_(PO_4_)_2_, Ca_2_P_2_O_7_ and (MgFe)_3_(PO_4_)_2_.8H_2_O were formed from the precipitation of PBS component and reside on the PLGA’s outermost surface, as postulated by previous study^63^. Also on the outer surface of PIPI sample, tightly aggregated and low soluble α-FeOOH was formed via the aging and crystallization of Fe(OH)_3_ which is resistant to mass transfer^60^. However, this outermost component will be more appreciably affected by the local acidic environment effect induced by the degradation of PLGA layer.

### Proposed degradation mechanism

In a neutral aqueous environment, generally iron (PPI) degrades via the following reactions:


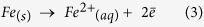



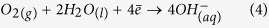



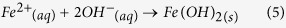


**Figure 9:**
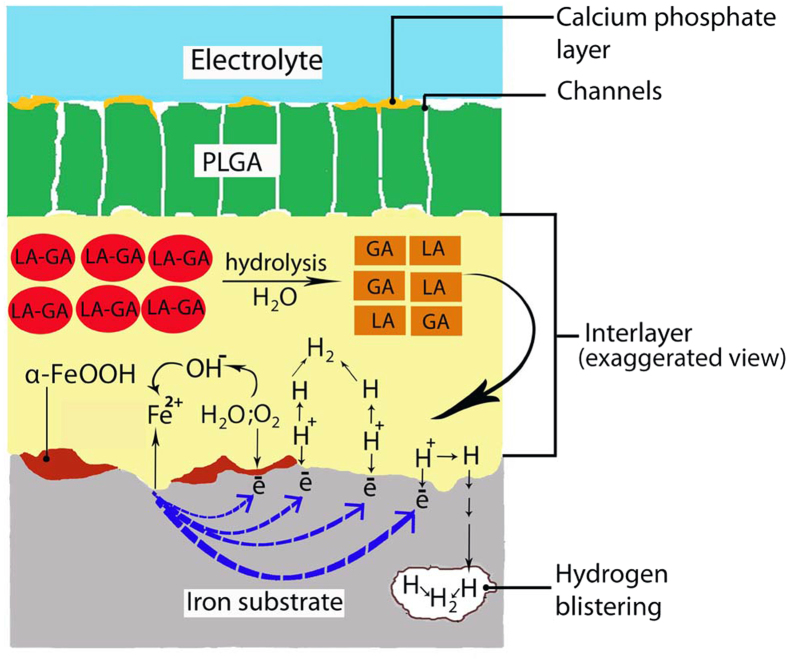
Proposed degradation mechanism at the iron/PLGA interface.

**Figure 10:**
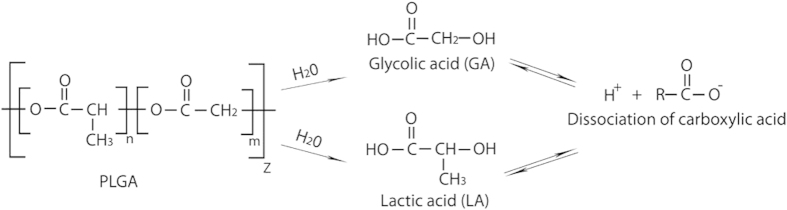
Hydrolysis reaction of the PLGA and dissociation of the carboxylic acid group.

The Fe(OH)_2_ could be further air-oxidized to form Fe_3_O_4_^60^ as indicated in XRD results (Fig. 8). Inadequate supply of oxygen due to the deposited Fe(OH)_2_ triggers the formation of Fe_3_O_4_ on the PPI surface. Hence, we can assume that some parts of the PPI's struts have been covered by tightly-adhered Fe(OH)_2_ which suppresses oxygen diffusion onto the iron surface. Further corrosion of iron is therefore inhibited as reported elsewhere^23^,^30^,^31^. Different situation occurred during the degradation of PCPI and PIPI samples. The strong interfacial interaction between the PLGA layer and the iron surface provoked continuous degradation of iron. A degradation mechanism for PCPI/PIPI is illustrated in Fig. 9. As the samples were immersed in the solution, PLGA degrade first through hydrolysis. This swift degradation created microspores throughout its surface and acted as channel for the electrolyte to permeate through and form an aqueous interlayer between the iron substrate and the PLGA layer. Formation of micropores on PLGA layer was proved by the reduction of pore resistance in previous impedance study^63^. The PLGA degrades into water-soluble fragments of lactic acid–glycolic acid (LA-GA) oligomers leading to dissociation of acid carboxylic end group (Fig. 10). The possible precipitation of calcium/phosphorus layer that can plug the microspores was hindered as the local acidic environment on the PLGA surface dissolve the layer. Calcium phosphate dissolution rate was accelerated in acidic environment owing to the PLGA degradation^79^,^80^. Similarly, dissolution occurred also on the formed Fe(OH)_2_ layer as hydroxide layers is more soluble in acidic environment^36^,^37^,^81^. The carboxylic acid group in the monomers (LA and GA) was further dissociated into H^+^ through a heterolytic cleavage of OH bond. The H^+^ ions react with the electrons at the iron surface to form hydrogen gas (hydrogen evolution) allowing a continuous passage of an equivalent quantity of Fe^2+^ into the solution. The hydrogen evolution disrupt the formation of inhibition layer facilitating oxygen diffusion onto the iron surface and enhance iron degradation^82^. Hence, the degradation of iron in PIPI is the sum of degradation triggered by both hydrogen evolution and oxygen reduction. These two electrons-consuming reactions have made up the total degradation of the iron and subsequently increase the overall corrosion rate of PIPI. The exchange current density of the H^+^ reduction is relatively one-order higher than that of the oxygen reduction (~10^−6^ and 10^−7^ A/cm^2^, respectively) for iron electrode^83^. Hence, the rate of cathodic reaction increase as H^+^ is reduced on the iron surface and more electrons are required to complete the reaction. Higher rate of electron supply requires higher rate of iron dissolution (Fe^2+^). The tendency of H^+^ to be reduced at the iron surface is more prominent as H^+^ ions are much easier to diffuse from the electrolyte onto the iron surface as the diffusion coefficient of hydrogen is bigger than oxygen^83^. In addition, hydrogen atoms formed during the reduction reaction may also absorbed into the iron substrate, form molecular hydrogen (H_2_) leading to hydrogen blistering and adding an extra aid to the overall iron degradation. Due to the concentration gradient (more hydrogen on the surface), the hydrogen atoms were adsorbed and absorbed and diffuses into the bulk of the metal^84^,^85^. An open circuit potential (OCP) experiment could indicate the charges residing on the metal surface^84^,^86^,^87^. Previous studies reported that the hydrogen absorption into bulk iron and stainless steel has decreased the corrosion potential toward more active region (more negative)^82^,^88^.

**Figure 11:**
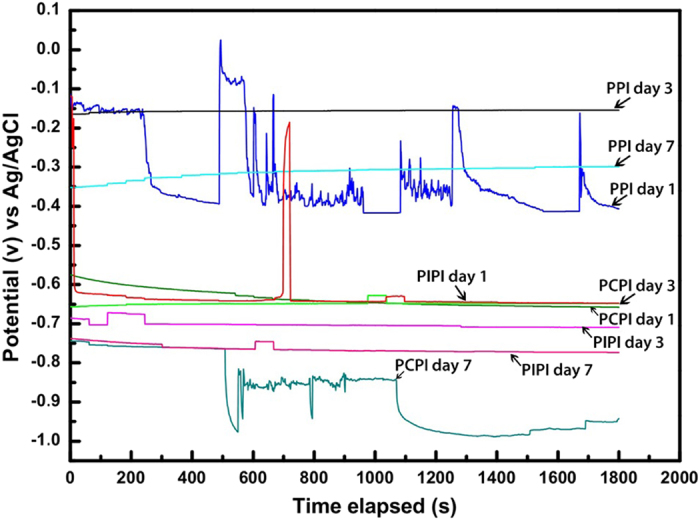
OCP results of PPI, PCPI and PIPI samples after day 1, 3 and 7 of immersion.

Figure 11 shows the open potential of PCPI and PIPI for day 1, day 3 and day 7 which are all below the potential at point of zero charge (pzc) of iron electrode (−0.48 V to −0.58 V/ Ag/AgCl) indicating the surface was negatively charged and had more tendency to attract cations^84^,^89^,^90^,^91^. At day 1 the open potential of PPI, PCPI and PIPI are about −0.42 V, −0.66 V and −0.65 V and shift to 0.30 V, −0.84 V, and −0.77 V in day 7 respectively. The shifting of the potentials to more negative values in PCPI and PIPI indicate the occurrence of dissolution on the iron surface over immersion time: meanwhile, passive oxide layers could form on PPI surface indicated by the upward (more positive) shifting^92^. The negatively-charged iron surface in PCPI and PIPI could provide a favorable condition to attract H^+^ and thus increases the hydrogen adsorption on the iron surface that consequently promotes its absorption into the bulk. As also proved by PDP result in Fig. 5c, the overall degradation of iron in PCPI and PIPI are predominantly controlled by the reduction of H^+^ ion (cathodically controlled). Hence, the more H^+^ attracted onto iron surfaces, the more tendency iron will degrade further. Strong interfacial interaction between PLGA and iron surface could be a significant catalyst for continuous H^+^ supply on iron surface through the hydrolysis of the PLGA.

### Cell viability

**Figure 12:**
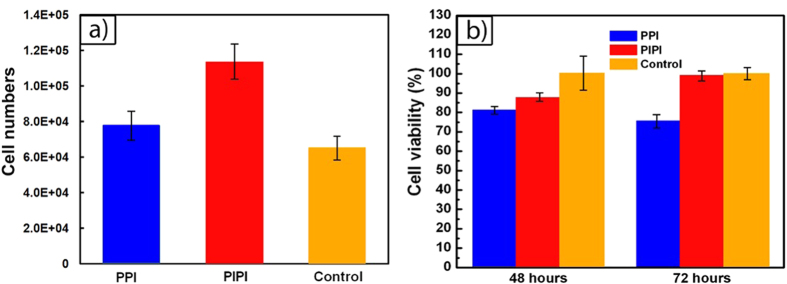
Results from in vitro HSF cells experiments: (**a**) cell count after 24 h of incubation, and (**b**) cell viability after 48 and 72 h of incubation in contact with PPI and PIPI samples.

Iron degradation has been known to support fibroblast cell viability^6^,^23^, however the combined effect with PLGA hydrolysis and iron degradation at a faster rate on the cells need to be verified, especially during the early degradation period. Figure 12 shows cell count and cell viability of human skin fibroblast (HSF) cells after exposure to the PPI and PIPI samples at different incubation time. Number of HSF cells is found higher for PIPI (~1.13 × 10^5^ cells) compared to PPI and control after 24 h incubation (Fig. 12a) while cell viability is also higher on the PIPI after 72 h incubation (Fig. 12b). These results indicate the supportive effect of PIPI degradation towards cell viability as it is known that PLGA surface provides a favorable microenvironment for cell proliferation^93^,^94^,^95^. Iron degradation, both *in vitro* and *in vivo*, has been also found beneficial effects toward cell viability, but a faster degradation is desirable^28^,^96^,^97^. The present preliminary cell viability study showed that the accelerated degradation of iron with the interaction with PLGA enhanced cell viability for an early degradation period up to 72 h. Even though this can represent the most active period of degradation, a longer incubation time is therefore required to observe the effect of a more steady degradation period towards the cells. Additionally, *in vivo* experiments are also suggested as *in vitro* degradation results are often different with that of *in vivo*.

## Conclusion

A new approach to accelerate the slow degradation of iron is successfully demonstrated by incorporating PLGA into porous pure iron (PPI) structure in the forms of PLGA-infiltrated porous iron (PIPI) and PLGA-coated porous iron (PCPI). The compressive strength of PCPI is higher than PIPI and PPI with the values fall within the range of that of cancellous bone. A stronger interfacial interaction between PLGA and iron surface resulted into a faster degradation of both PCPI and PIPI. Degradation rate of PIPI is faster than PCPI and PPI as measured by weight loss and Fe^2+^ release and supported by results from potentiodynamic polarization, potentiostatic polarization and electrochemical impedance spectroscopy. The fast degradation rate of PCPI and PIPI is greatly incited by the hydrolysis of PLGA that produced soluble monomers consisting of carboxylic acid groups. The hydrolysis is also suspected to induce hydrogen evolution in addition to oxygen reduction and further accelerates the degradation via two electron-consuming cathodic reactions. The fast degradation and the interaction with PLGA maintained a good fibroblast cell viability during the early and most active degradation period.

## Experimental Section

### Sample preparation

Two sets of porous pure iron (PPI) sheets (thickness: porosity: pore size = 1.6 mm: 88%: 450 μm and 2.5 mm: 92%: 800 μm, Alantum, Korea) and poly(DL-lactic-*co*-glycolic acid) (PLGA) granules (50:50, inherent viscosity = 0.38 dl/g, Lactel, USA) were used as the raw materials. The PLGA granules were dissolved in chloroform to produce PLGA solution (5% *w/v*). The PLGA solution was infiltrated into the PPI at vacuum pressure of −0.07 MPa for 20 min to produce samples of fully dense PLGA-infiltrated porous iron (PIPI). Samples of partially dense PLGA-coated porous iron (PCPI) were produced by dipping the PPI into the PLGA solution using a dip coating machine (Dip Coater PTL-MMB, MTI Corp, USA) at down and withdraw speeds of 200 mm/min for 1 cycle at dwelling time of 8 s. The residual solvent on the samples were let to evaporate at room temperature for 48 h.

### Microstructure and interfacial characterization

Microstructural analysis and elemental mapping were done on the polished and gold coated PIPI and PCPI samples using a scanning electron microscope (SEM, TM3000, Hitachi, Japan) coupled with an energy-dispersive X-ray spectroscopy (EDS, SwiftED 3000, Oxford Instrument, UK). Prior to the EDS mapping, the PIPI were cross-sectioned and mechanically ground using silicon carbide SiC papers up to 1200 grit. The interfacial interaction between the iron surface and the PLGA was investigated using an attenuate transform infrared-Fourier transform infrared spectroscopy (ATR-FTIR, Nicolet iS5, Thermo Scientific, USA) and further confirmed by a thermogravimetric analyzer and differential thermal analyzer (TGA-DTA, TGA/SDTA85, Mettler, USA) and an X-ray photoelectron spectroscopy (KRATOS Axis Ultra, Kratos Analytical, UK). The TGA-DTA analysis were run from room temperature to 700 °C at a heating rate of 10 °C/min in nitrogen environment with a purging rate of 10 ml/min. The XPS analysis was conducted using an incident X-ray radiation Mg Kα_1,2_ = 1253.6 eV at vacuum pressure of 5 × 10^−9^ torr. Narrow multiplex scans were recorded with 29.35 eV and 0.1 eV step size. The spectra obtained on the PLGA and PIPI samples were shifted and calibrated to set the C-C/C-H peak components of the C_1s_ peak at a binding energy of 284.8 eV to correct the sample charging. This peak at 284.8 eV of C-C/C-H component was used as the reference peak for the entire spectrum^98^,^99^,^100^. Data analysis and the curve fitting of C_1s_ and O_1s_ peaks were evaluated using the CasaXPS software (version 2.3.16, Casa Software Ltd., UK) and the decomposition were done by the Shirley-type background removal.

### Mechanical testing

Compressive tests were done on five samples of 450 μm and 800 μm PPI, PIPI and PCPI (specimen dimension = 15 × 12.5 × 1.6 mm) at 0.001 mm/s cross-head speed with a 1 kN load cell using a universal testing machine (Instron 8874, Instron, USA). The test and the determination of compressive strength, yield strength and modulus of elasticity were done by following the ASTM D1621 and ISO 844 standards. The compressive strengths were computed as the first maximum on the stress-strain curve while the yield strengths were determined at 0.2% offset strain. The values of modulus of elasticity were measured as the slopes of stress-strain curves in the elastic deformation region, while toughness was determined by calculating the area under the compression curves.

### Degradation testing

Degradation tests were carried out by using five different approaches: weight loss complimented with ion release measurement as a direct indicator of degradation rate, then potentiodynamic polarization (PDP), potentiostatic polarization (PSP) and electrochemical impedance spectroscopy (EIS) to measure corrosion current and the effect of PLGA on the charge transfer. Static immersion test was performed on PPI, PCPI and PIPI samples where each 5 specimens (dimension = 6 × 6 × 1.6 mm) were immersed in 30 ml of phosphate buffered solution (PBS, Sigma Aldrich, USA) in a 50 ml glass beaker. The temperature and pH were monitored and maintained at 37 ± 0.5 °C and 7.4 by placing the beakers in an isothermal water bath and by refreshing the solution every 24 h. Weight loss was measured at week 1, 2 and 4 on at least 5 specimens from each group. The specimens were rinsed in deionized water and ethanol then brushed gently as specified by the ASTM G1 standard, followed by air and vacuum drying for 48 h to completely remove all degradation products before weighing. Degradation rate was calculated by using the following formula:


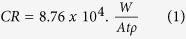


where CR = corrosion rate (mm/year), W = weight loss (g), A = area (cm^2^), t = time of exposure (h) and ρ = density (g/cm^3^). The density was determined via Archimedes principle method where the values of 1.89 g/cm^3^, 1.12 g/cm^3^ and 1.26 g/cm^3^ for PPI, PCPI and PIPI respectively, were used.

The PDP tests were carried out using a standard three-electrode system in 250 mL of PBS with graphite rod as the auxiliary and Ag/AgCl (KCl 3.5 M) as the reference electrodes connected to a potentiostat (Versastat 3, Princeton Applied Research, USA). The working electrode (5 specimens of each PPI, PCPI and PIPI samples) has 0.502 cm^2^ exposed area. The tests were conducted at 37 ± 0.5 °C with a scan rate of 1.0 mV/s over a potential range of −0.25 V to 0.25 V after a stable open circuit potential (OCP) was obtained. Corrosion current was determined using Tafel extrapolation and corrosion rate were measured using the following formula:

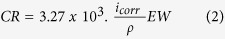
where *i*_*corr*_ = corrosion current density (μA/cm^2^), EW = equivalent weight (28 g/eq) and ρ = density.

The PSP tests were conducted using similar instrument set-up and set of specimens as for the PDP tests but by using a constant stepping potential of −0.5 V for 18 hours after 7 days immersion in 250 mL PBS solution at 37 ± 0.5 °C and the trends in the current densities were monitored. Meanwhile, the EIS experiments were performed on similar set of specimensas for the PDP tests up to 2 hours in 250 mL PBS at room temperature. VersaStudio software (Princeton Applied Research, USA) was used for data acquisition at open circuit potential over a frequency range of 100 kHz down to 10 mHz using a peak-to-peak 10 mV sinusoidal perturbation. Additional electrochemical tests were conducted to evaluate the susceptibility of hydrogen atom adsorption onto iron surface by using open circuit potential (OCP) method. Similar instrument set-up and set of specimens as for the PDP tests was used and the tests were conducted at 37 ± 0.5 °C for 30 minutes in 250 mL PBS after day 1, day 3 and day 7 of immersion.

### Ion concentration, surface morphology and degradation product analysis

Samples of 6 ml of the test solution from each group after immersion tests were analyzed using a graphite furnace atomic absorption spectrophotometer (GF-AAS, AAnalyst 400, Perkin Elmer, USA) to determine ferrous ion (Fe^2+^) concentration. Surface morphology of the degraded samples was analyzed using the SEM/EDS after week 1 and 4 of immersion. Elemental composition profile across the iron’s substrate and degradation layer was mapped using EDS profiling while an X-ray diffractometer (XRD, Bruker, USA) was used to determine the phases residing in the 4 week degraded samples. The XRD data were analyzed using X’Pert HighScore software (PANalytical, Amelo, Netherland). Fourier transform infrared spectroscopy (FTIR, Nicolet iS5, Thermo Scientific, USA) was used to characterize the changes in chemical structure of degraded samples after week 2 and 4 of immersion. The FTIR absorbance spectra were obtained with 32 scans per sample over the range of 4000 to 400 cm^−1^ and then subtracted from the background ratio and plotted using OriginPro (OriginLab, USA). The FTIR was also used to ascertain the formation of common degradation products of iron and the changes in O-H and carboxylic end group of the degraded PCPI and PIPI samples.

### Cell viability test

Human skin fibroblast cells (HSF 1184) were cultured in minimum essential medium (MEM) containing 10% fetal bovine serum and 1% penicillin/streptomycin at 37 °C in a 5% (v/v) CO_2_ incubator. As much as 2 × 10^5^ cells/ml was seeded directly onto PPI, PIPI and control specimens as used for the immersion test (surface area 1.5 × 1.5 mm^2^) and placed in a 6-well plate. Possible effect of chlorinated solvent residue, which may inhibit cell proliferation as found in our preliminary studies, was diminished by continuous stirring of PLGA solution during PIPI preparation to evaporate all the remaining solvent. The control consists of seeded cells and medium only without specimens. Six replicates were used for each group. Two mL of MEM enriched with 10% fetal bovine serum was added to each well. Cultures were maintained in the incubator up to 72 h for cell attachment analysis while cell adhesion was investigated after 24 h. Subsequently, cell proliferation analysis after 24 and 72 h were also performed using MTT (Invitrogen, USA). Five mg/ml MTT (3-(4,5-dimethylthiazol-2-yl)-2,5-diphenyl tetrazolium) solution was prepared by dissolving MTT in PBS. 100 μl MTT solution was added to each well to form formazan by mitochondrial dehydrogenase. After 4 h of incubation, 1 ml of DMSO was added and shaken gently for 10 min to dissolve the formazan crystals. 200 μl of supernatant was transferred to 96-well plates and three data points were obtained from each sample. The optical density was determined with a microplate reader (Multiskan FC, Thermo Scientific, USA) at a wavelength 570 nm. All results were expressed as mean ± standard deviation (SD) of 6 replicates. The SigmaPlot^TM^ software (Systat Software, USA) and student’s t-test was used for statistical analysis and multiple comparison and significance level was determined at p < 0.05.

## Additional Information

**How to cite this article**: Yusop, A. H. M. *et al*. Controlling the degradation kinetics of porous iron by poly(lactic-*co*-glycolic acid) infiltration for use as temporary medical implants. *Sci. Rep*. **5**, 11194; doi: 10.1038/srep11194 (2015).

## Figures and Tables

**Table 1:** Peak deconvolution of C_1s_ and O_1s_ corresponding to C=O component of the PLGA and PIPI.

**Sample**	**C_1s_**		**O_1s_**	
	**BE (eV)**	**FWHM (eV)**	**BE (eV)**	**FWHM (eV)**
PLGA	288.31	1.68	532.44	1.90
PIPI	289.03	1.68	532.97	1.90

Note: BE = binding energy, FWHM = full width at half maximum.

**Table 2:** Mechanical properties of PPI, PCPI and PIPI samples.

**Samples**	**Yield strength (MPa)**		**Compressive strength (MPa)**		**Elastic modulus (MPa)**		**Toughness (J.m^−3^)**	
	**450 μm**	**800 μm**	**450 μm**	**800 μm**	**450 μm**	**800 μm**	**450 μm**	**800 μm**
PPI	0.22 ± 0.04	0.16 ± 0.01	0.28 ± 0.02	0.20 ± 0.01	3.93 ± 0.21	3.88 ± 0.31	1955.7	1090.8
PCPI	0.65 ± 0.08	0.27 ± 0.02	0.71 ± 0.04	0.30 ± 0.02	14.22 ± 1.10	6.15 ± 1.52	5858.8	1890.3
PIPI	0.38 ± 0.02	0.20 ± 0.01	0.42 ± 0.01	0.24 ± 0.01	8.78 ± 0.23	4.17 ± 0.53	5983.5	2079.4

**Table 3:** Impedance parameters of PPI, PCPI and PIPI samples.

**Samples**	**R_ct_ (Ω.cm^2^)**	**f_max_ (Hz)**	**|Z| (Ω.cm^2^)**	**C_dl_ (μF.cm>^−1^)**
PPI	3250 ± 48	1.26	866.74	38.89
PCPI	1250 ± 200	0.20	600.04	636.63
PIPI	275 ± 30	0.05	511.98	11347.95

Note: R_ct_ = charge transfer resistance, f_max_ = frequency at which the imaginary component of the impedance, Z_im_, is maximum, C_dl_ = double layer capacitances = (2πf_max_R_ct_)^−1^.
